# Retraction: LncRNA OIP5-AS1 contributes to ox-LDL-induced inflammation and oxidative stress through regulating the miR-128-3p/CDKN2A axis in macrophages

**DOI:** 10.1039/d1ra90042k

**Published:** 2021-01-28

**Authors:** Laura Fisher

**Affiliations:** Royal Society of Chemistry Thomas Graham House, Science Park, Milton Road Cambridge CB4 0WF UK advances-rsc@rsc.org

## Abstract

Retraction of ‘LncRNA OIP5-AS1 contributes to ox-LDL-induced inflammation and oxidative stress through regulating the miR-128-3p/CDKN2A axis in macrophages’ by Xiaojuan Li *et al.*, *RSC Adv.*, 2019, **9**, 41709–41719, DOI: 10.1039/C9RA08322G.

The Royal Society of Chemistry hereby wholly retracts this *RSC Advances* article due to concerns with the reliability of the data. The paper was analysed by experts who fact-checked the identities of the described nucleotide sequence reagents,^1^ and found errors with the following nucleotide sequence reagents reported in the article: OIP5-AS1 forward and reverse primers, miR-128-3p forward and reverse primers, CDKN2A forward and reverse primers, and U6 reverse primer. These incorrectly described RT-PCR primers affect the reliability of the results presented in Fig. 1, 5, 6 and 7. Furthermore, the reported U6 reverse primer is non-targeting, and given that U6 is used as a control, all the reported RT-PCR results are unreliable.

The authors were asked to provide the raw data for this article, but did not respond. Given the significance of the concerns about the validity of the data, and the lack of raw data, the findings presented in this paper are not reliable.

The authors have been informed but have not responded to any correspondence regarding the retraction.

Signed: Laura Fisher, Executive Editor, *RSC Advances*.

Date: 15^th^ January 2021.

## Supplementary Material
